# A phase II study of talazoparib monotherapy in patients with wild-type *BRCA1* and *BRCA2* with a mutation in other homologous recombination genes

**DOI:** 10.1038/s43018-022-00439-1

**Published:** 2022-10-17

**Authors:** Joshua J. Gruber, Anosheh Afghahi, Kirsten Timms, Alyssa DeWees, Wyatt Gross, Vasily N. Aushev, Hsin-Ta Wu, Mustafa Balcioglu, Himanshu Sethi, Danika Scott, Jessica Foran, Alex McMillan, James M. Ford, Melinda L. Telli

**Affiliations:** 1 Department of Internal Medicine and Cecil H. and Ida Green Center for Reproductive Biology Sciences, University of Texas Southwestern Medical Center, Dallas, TX USA; 2 Department of Medicine, University of Colorado, Aurora, CO USA; 3 Myriad Genetics, Salt Lake City, UT USA; 4 Department of Medicine, Stanford University School of Medicine, Palo Alto, CA USA; 5 Natera, Austin, TX USA; 6 Department of Statistics, Stanford University School of Medicine, Palo Alto, CA USA; 7 Department of Genetics, Stanford University School of Medicine, Palo Alto, CA USA

**Keywords:** Breast cancer, Cancer genetics, Cancer, Cancer therapy

## Abstract

Talazoparib, a PARP inhibitor, is active in germline *BRCA1* and *BRCA2* (g*BRCA1*/*2*)-mutant advanced breast cancer, but its activity beyond g*BRCA1*/*2* is poorly understood. We conducted Talazoparib Beyond BRCA (NCT02401347), an open-label phase II trial, to evaluate talazoparib in patients with pretreated advanced HER2-negative breast cancer (*n* = 13) or other solid tumors (*n* = 7) with mutations in homologous recombination (HR) pathway genes other than *BRCA1* and *BRCA2*. In patients with breast cancer, four patients had a Response Evaluation Criteria in Solid Tumors (RECIST) partial response (overall response rate, 31%), and three additional patients had stable disease of ≥6 months (clinical benefit rate, 54%). All patients with germline mutations in *PALB2* *(*g*PALB2*; encoding partner and localizer of BRCA2) had treatment-associated tumor regression. Tumor or plasma circulating tumor DNA (ctDNA) HR deficiency (HRD) scores were correlated with treatment outcomes and were increased in all g*PALB2* tumors. In addition, a g*PALB2*-associated mutational signature was associated with tumor response. Thus, talazoparib has been demonstrated to have efficacy in patients with advanced breast cancer who have g*PALB2* mutations, showing activity in the context of HR pathway gene mutations beyond g*BRCA1*/*2*.

## Abstract (web summary)

Telli and colleagues present a phase II clinical trial of the PARP inhibitor talazoparib in patients with solid tumors and show that the drug has activity in patients with breast cancer with mutations in other homologous recombination pathway genes beyond *BRCA1* and *BRCA2*.

## Main

PARP inhibitors, including talazoparib and olaparib, are currently approved for the treatment of HER2-negative advanced breast cancer in patients who harbor a genomic *BRCA1* and/or *BRCA2* (g*BRCA1*/*2*) mutation^1,2^. Additional drugs in this class, including niraparib and rucaparib, are approved for ovarian cancer therapy or maintenance^3^. These therapies are rooted in the concept of synthetic lethality wherein deficiency in *BRCA1* and/or *BRCA2* or other homologous recombination (HR)-associated genes in the tumor renders tumor cells sensitive to PARP inhibition by disabling a PARP-dependent single-stranded DNA (ssDNA) repair pathway in the context of defective HR DNA repair, which is dependent on *BRCA1,* *BRCA2* and associated factors^4^. Thus, in the setting of g*BRCA1*/*2* mutation (or presumably somatic loss of heterozygosity (LOH) for *BRCA1 or* *BRCA2*) and PARP inhibitor therapy, tumor cells cannot repair ssDNA breaks that accumulate through metabolic processes and DNA replication leading to tumor-specific lethality, whereas normal cells can use HR to repair these lesions and thus are spared. This observation has driven the clinical development of PARP inhibitors for tumors with g*BRCA1*/*2* mutations.

The OlympiAD^5^ and EMBRACA^6^ clinical trials of olaparib and talazoparib, respectively, in g*BRCA1*/*2*-mutant advanced metastatic breast cancer showed similar increases in progression-free survival (PFS) of approximately 3 months compared to chemotherapy treatment of physician’s choice, which led to Food and Drug Administration (FDA) approval of these agents for this specific patient population. Efficacy of monotherapy PARP inhibitors has also been demonstrated in patients with pancreatic cancer with g*BRCA1*/*2* mutations (POLO trial^7^), as well as in patients with prostate cancer with either g*BRCA1*/*2* mutations (PROfound^8^) or other HR-associated mutations (TOPARP-A/B^9,10^, TRITON-2 (refs. ^11,12^), GALAHAD^13^, TALAPRO-1 (ref. ^14^)). Recently, veliparib has also shown efficacy in combination with first-line chemotherapy for advanced ovarian cancer followed by maintenance therapy (VELIA/GOG-3005)^15^. Veliparib also has benefit if used in combination with chemotherapy in advanced HER2-negative breast cancers with g*BRCA1*/*2* mutations (BROCADE3)^16^. These studies highlight the importance of biomarkers in selecting patients for PARP inhibitor therapy.

Possible biomarkers explored for PARP inhibitor-based therapy include germline DNA alterations, somatic (tumor) DNA mutations and HR deficiency (HRD) scores. Large population-based studies have identified that 24% of patients with breast cancer and 30.9% of patients with ovarian cancer have undergone germline DNA analysis for mutations associated with cancer onset in the current era^17^. These numbers are expected to increase as DNA testing continues to expand into community-based oncology practices. This type of testing has the potential to identify g*BRCA1*/*2* mutations or other germline HR-associated gene mutations that could be used to select PARP inhibitor therapy for patients with advanced disease.

Similar to germline testing, somatic tumor genetics is rapidly expanding, especially in the treatment of advanced or metastatic cancers, with the goal of identifying mutations associated with therapeutic benefit of FDA-approved agents or as enrollment criteria for clinical trials of novel or expanded-access therapies. In one large retrospective analysis of over 17,000 tumors across 21 different tumor types, we found that 17.4% of tumors harbored mutations in genes associated with HR pathways^18^. In primary prostate cancers, the prevalence of HRD mutations was 9.9%^19^, with a higher rate (11.8–21.3%) found in metastatic lesions^20,21^. This suggests that PARP inhibitors could benefit patients with a wider subset of tumors than currently envisioned by only assessing g*BRCA1*/*2* status.

As opposed to assessing gene-specific mutations in *BRCA1* and *BRCA2* or other HR-associated genes, HRD scores can tabulate a genome-wide metric of HRD. This metric can integrate up to three types of chromosomal aberrations associated with HRD, including LOH, telomeric allelic imbalance and large-scale state transitions^22^. Clinical studies have determined that an elevated HRD score is associated with response to platinum-based chemotherapy in early-stage triple-negative breast cancer^22^ and increased PFS in ovarian cancer treated with niraparib^23^. Additionally, higher LOH was associated with benefit from rucaparib maintenance therapy (ARIEL 3)^24^. A high HRD score may reflect the presence of *BRCA1* and/or *BRCA2* mutations, mutations in other HR-associated genes or methylation of HR genes. Therefore, tumors with high HRD scores are a promising subset to consider for PARP inhibitor therapy.

While HRD and LOH assays have been approved by the FDA to select patients with ovarian cancer for treatment with PARP inhibitors, the data in advanced breast cancer are less clear. The Treating to New Targets trial did not demonstrate a role for HRD testing to identify patients with wild-type *BRCA1* and *BRCA2* who have advanced triple-negative breast cancer more likely to benefit from carboplatin as opposed to taxane chemotherapy^25^. However, in the SWOG 1416 trial of cisplatin with or without veliparib, an higher HRD score was associated with improved PFS with the addition of veliparib to cisplatin in patients with germline wild-type g*BRCA1*/*2* advanced triple-negative breast cancer^26^.

Taken together, previous studies have suggested that other biomarkers beyond g*BRCA1*/*2* may be associated with clinical responses to PARP inhibitors, but the data remain relatively sparse. We explored the hypothesis that triple-negative breast cancers with high HRD scores (cohort A) or any solid tumor with germline or somatic mutations in HRD-associated genes other than *BRCA1* and *BRCA2* (cohort B) could be used to select patients for talazoparib monotherapy in the Talazoparib Beyond BRCA phase II clinical trial. Here we report the results of 20 patients treated in cohort B who were enrolled based on identification of an HR pathway-associated mutation other than g*BRCA1*/*2* on either germline or somatic next-generation sequencing (NGS) testing. We report that patients with breast cancer with mutations beyond g*BRCA1*/*2* had a 31% overall response rate (ORR), whereas no objective responses were observed in non-breast tumors. The responses of patients with breast cancer were driven, in part, by patients with germline mutations in *PALB2* *(*g*PALB2*; encoding partner and localizer of BRCA2) and were correlated with high HRD scores. These results indicate that PARP inhibitors should be further explored in metastatic or advanced breast cancers that have HR-associated mutations beyond *BRCA1* and *BRCA2*.

## Results

### Patient characteristics

**Extended Data Fig. 1:**
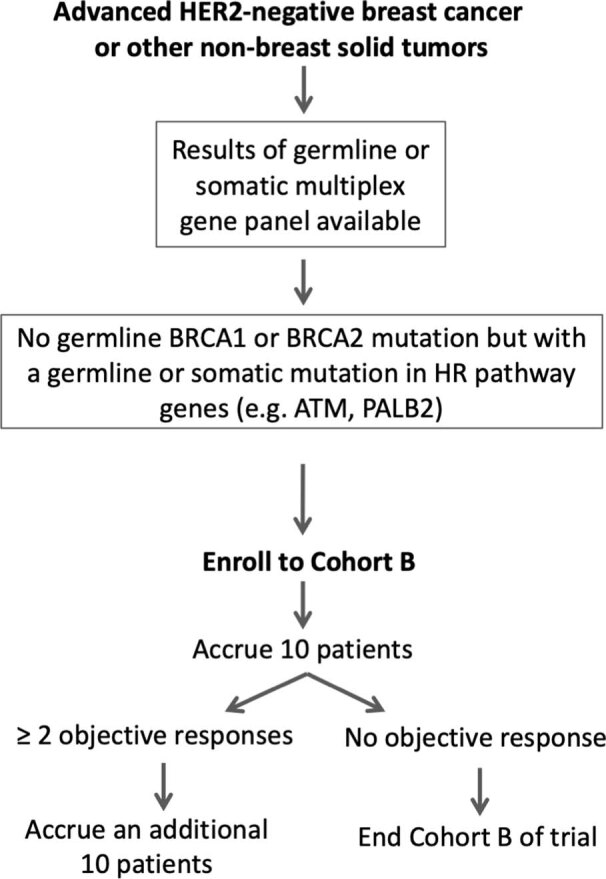
Talazoparib Beyond BRCA study schema for cohort B. Cohort B enrolled patients with advanced HER2-negative breast cancer or other solid tumors lacking a germline BRCA1 or BRCA2 mutation with either a germline or somatic mutation in a gene associated with HR-deficiency. Patients were treated with single-agent talazoparib in an initial cohort of 10 patients. On the basis of observed responses in the first 10 patients, an additional 10 patients were enrolled and treated.

Talazoparib Beyond BRCA is an open-label, non-randomized single-institution phase II trial that enrolled patients who had undergone either germline genetic testing or somatic tumor multiplex gene testing and who had demonstrated a pathogenic or likely pathogenic mutation in an HR-associated gene (Extended Data Fig. 1 and Supplementary Table 1). Patients with germline or somatic *BRCA1* or *BRCA2* mutations were excluded. Eligible patients had metastatic or recurrent HER2-negative breast cancer or another solid tumor with previous progression of disease on at least one line of therapy for metastatic/advanced disease; there was no upper limit on the number of previous therapies. The primary objective was the ORR and secondary objectives included determination of the clinical benefit rate (CBR), PFS and safety.

Twenty patients were consented and enrolled between August 2015 and December 2018. All patients initiated therapy with talazoparib and received at least one cycle of therapy. Based on two instances of partial responses (PRs) observed in the first stage of ten patients, an additional ten patients were enrolled according to the study design (Extended Data Fig. 1). Of the 20 patients enrolled, 13 patients had HER2-negative breast cancer (*n* = 11 hormone receptor positive and *n* = 2 triple-negative breast cancer) and 7 patients had other tumor types (*n* = 3 pancreatic cancer and *n* = 1 each of colon cancer, mixed Mullerian uterine cancer, testicular cancer and parotid acinic cell carcinoma) as shown in Table 1. Most of the patients were female (75%) with a median age of 53.9 years. Patients had received a median of two prior lines of therapy for advanced disease (range, 1–8). Prior lines of therapy included chemotherapies, hormonal therapies and targeted agents. Platinum-based therapies had been previously administered to 35% of patients, but patients with disease progression within 8 weeks of the last platinum dose were excluded from this study.

**Table 1:** Baseline characteristics and mutations used for enrollment of patients treated in cohort B

Characteristic		*n* = 20
**Sex**		
Female		15 (75%)
Male		5 (25%)
**Age (years)**		
Median		53.9
Range		49–80
**Cancer type**		
Breast		13 (65%)
HR+/HER2-		11 (55%)
ER-/PR-/HER2-		2 (10%)
Pancreas		3 (15%)
Colon		1 (5%)
Mixed Mullerian uterine		1 (5%)
Testicular		1 (5%)
Parotid acinic cell carcinoma		1 (5%)
**Prior lines of therapy for advanced disease**		
Median		2
Range		1–8
**Prior platinum**		
Yes		7 (35%)
No		13 (65%)
**Mutation**	**Germline (*n*** = 15)	**Somatic (*n*** = 9)
*ATM*	3	2
*ATR*	0	1
*BRIP1*	2	0
*CHEK2*	3	0
*FANCA*	1	0
*PALB2*	6	0
*PTEN*	0	5
*RAD50*	0	1

HR, hormone receptor; ER, estrogen receptor; PR, progesterone receptor.

Enrolled patients had germline pathogenic or likely pathogenic mutations in *ATM* (*n* = 3), *BRIP1* (*n* = 2), *CHEK2* (*n* = 3), *FANCA* (*n* = 1) and *PALB2* (*n* = 6) or somatic mutations in *ATM* (*n* = 2), *ATR* (*n* = 1), *PTEN* (*n* = 5) and *RAD50* (*n* = 1) as detected by any CLIA-approved NGS assay performed on either germline tissue or tumor tissue (Table 1). Two patients had multiple qualifying mutations at the time of study enrollment (pancreatic cancer with g*PALB2* and g*BRIP1* mutations and breast cancer with g*CHEK2,* g*FANCA* and s*PTEN mutations*).

### Talazoparib efficacy

**Fig. 1:**
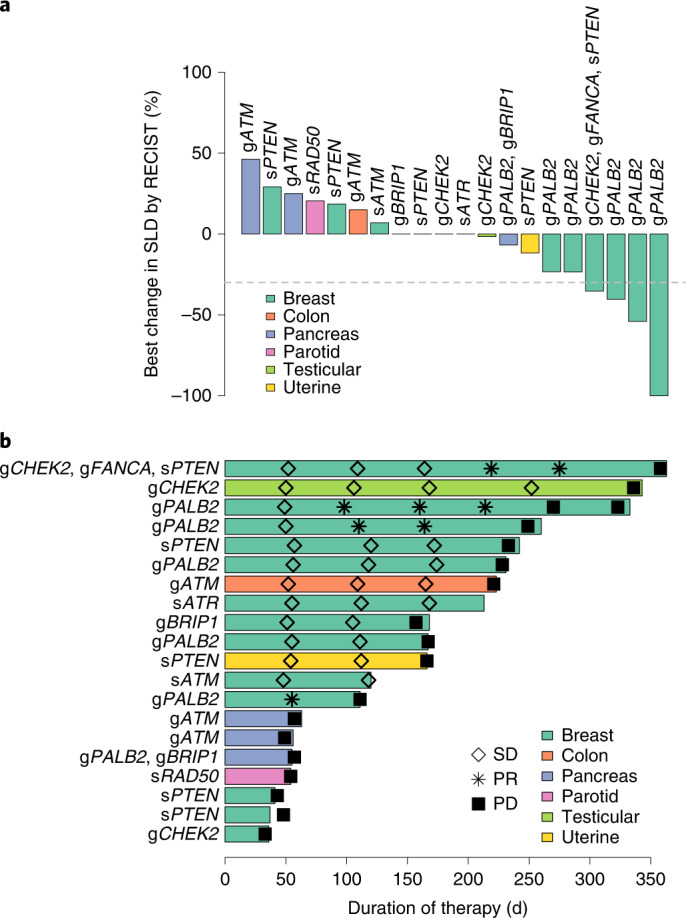
Treatment response and duration for therapy for all patients. **a**, Waterfall plot of best change in the sum of longest diameters (SLD) of target lesions by RECIST v.1.1 for all patients (*n* = 20) treated in cohort B, colored by tumor type. Germline (g) or somatic (s) mutations in genes used for enrollment are indicated. The dashed line represents a 30% decrease in tumor size. **b**, Duration of therapy by swimmers plot indicating duration of talazoparib therapy. Tumor responses assessments are noted. *n* = 20 patients. Source data

All enrolled patients were treated with talazoparib monotherapy at the FDA-approved dose of 1 mg orally daily. All patients had discontinued therapy by 17 April 2019. Nineteen patients discontinued therapy due to disease progression; one patient withdrew from therapy with Response Evaluation Criteria in Solid Tumors (RECIST) stable disease (SD) due to concern of nontarget disease enlargement. Among all enrolled patients, the best ORR was 20% (95% confidence interval (CI), 6–44%) and CBR was 45% (95% CI, 23–68%). Response rates were also stratified into groups with breast cancer and non-breast cancer (Table 2). Among the 13 patients with breast cancer, 4 patients (31%) achieved a PR as best response; 6 patients (46%) had SD as best response, and 3 patients (23%) had progressive disease (PD) as their best response. This led to an ORR of 31% (95% CI, 9–61%) and a CBR of 54% (95% CI, 21–81%) in patients with breast cancer. Among the non-breast cancer cohort (*n* = 7), no responses were observed, but 4 patients (57%) had SD as their best response, while 3 patients (43%) had PD as their best response. Thus, the non-breast cancer cohort had a CBR of 29% (95% CI, 37–71%). Among the 6 patients with g*PALB2* mutations, the ORR was 50% (95% CI, 19–81%). Best treatment responses are summarized by waterfall plot in Fig. 1a for all 20 patients treated in the trial.

**Table 2:** Best responses by RECIST v.1.1

Best response	Response rate, *n* (%)		
	Breast cancer (*n* = 13)	Non-breast cancer (*n* = 7)	Combined (*n* = 20)
CR	0 (0%)	0 (0%)	0 (0%)
PR	4 (31%)	0 (0%)	4 (20%)
SD	6 (46%)	4 (57%)	10 (50%)
PD	3 (23%)	3 (43%)	6 (30%)
ORR (CR + PR)	4 (31%; 95% CI, 9–61%)	0 (0%; 95% CI, 0–41%)	4 (20%; 95% CI, 6–44%)
CBR (CR + PR + SD for ≥ 6 months)	7 (54%; 95% CI, 21–81%)	2 (29%; 95% CI, 37–71%)	9 (45%; 95% CI, 23–68%)

Patients were divided into breast cancer and non-breast cancer subgroups. ORR includes confirmed and unconfirmed responses.

Duration of time on therapy was assessed (Fig. 1b). Across all 20 treated patients, the median time on therapy was 23.8 weeks, which was longer in the breast cancer subgroup compared to the non-breast cancer subgroup (median of 24.0 versus 12.4 weeks, respectively). Five patients with SD as their best response remained on therapy for more than 30 weeks, including a patient with testicular cancer with a g*CHEK2* mutation, a patient with colon cancer with a g*ATM* mutation and three patients with breast cancer with s*PTEN*, g*PALB2* or s*ATR* mutations. Of the four patients who achieved a PR as their best response, three remained on therapy for >35 weeks. The median PFS was 5.6 months (95% CI, 3.6–7.6) among the participants with breast cancer, 2.6 months (95% CI, 0–5.3) among the participants with non-breast cancer and 5.5 months (95% CI, 3.9–7.1) in the combined cohort. Among the patients with a g*PALB2* mutation, the median PFS was 6.9 months (95% CI, 4.4–9.4).

### Safety

Treatment-related toxicities associated with talazoparib monotherapy in this study were manageable and similar to the previously documented experiences with this agent (Supplementary Table 2). Treatment-related hematologic adverse events of any grade were experienced by 55% of the patients, whereas 30% had a grade 3 hematologic adverse event. No grade 4 or 5 hematologic adverse events were observed. Five patients required a dose reduction for hematologic toxicity. Three patients required red blood cell transfusions during treatment, and two patients required platelet transfusions.

Seventy percent of the patients experienced a non-hematologic toxicity of any grade. Grade 3 non-hematologic adverse events were rare (one patient with grade 3 fatigue). Overall, nausea and fatigue were the most common treatment-related non-hematologic adverse events experienced by 45% and 20% of the patients, respectively (all grades). No patient required permanent drug discontinuation as a result of an adverse event.

### Evaluation of tumor HRD score as a biomarker for talazoparib response

**Fig. 2:**
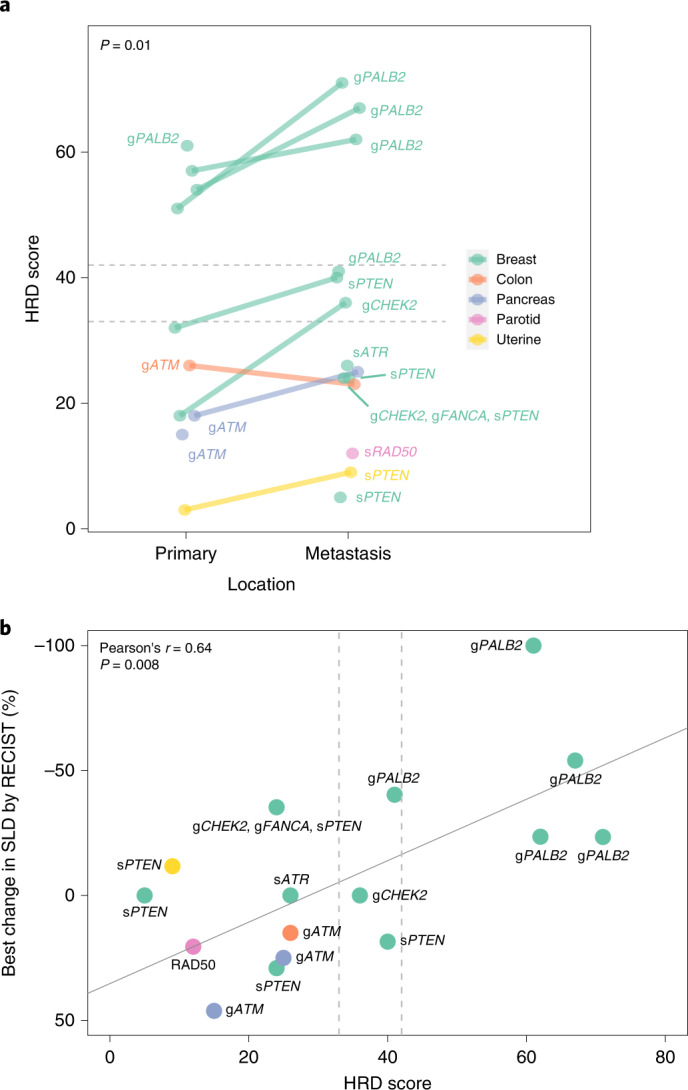
Tumor HRD scores and correlation with treatment response. HRD score was obtained by NGS assay (Myriad myChoice) from tumor biopsies (*n* = 15 patients; 2 patients excluded for insufficient sample and 2 patients excluded for assay failure). **a**, HRD score is plotted in primary and metastatic samples for all evaluable patients with lines connecting paired samples. Horizontal dashed lines indicate an HRD score threshold of ≥33 (captures 99% of known *BRCA1*- and/or *BRCA2*-deficient ovarian cancers) or ≥42 (captures 95% of known *BRCA1*- and/or *BRCA2*-deficient ovarian cancers). Significance testing by paired *t*-test for primary versus metastatic HRD scores. **b**, Correlation of HRD score with talazoparib treatment response. HRD score is plotted against best change in SLD by RECIST. Pearson’s correlation is indicated by the solid line, with associated *r* and two-sided *P* value denoted. Vertical dashed lines indicate an HRD threshold of ≥33 or ≥42. In cases where patients had more than one HRD score (for example, due to assay of primary and metastatic tumors), the higher score was used. Source data

To determine whether the tumors from patients enrolled on this study with mutations beyond g*BRCA1*/*2* also showed high levels of genomic instability, we performed the Myriad myChoice HRD CDx assay (Fig. 2a) on primary (*n* = 10) or metastatic (*n* = 14) formalin-fixed paraffin-embedded (FFPE) tumor tissue of 18 of the 20 patients treated on this trial (2 patients were excluded for insufficient sample). Eight patients had HRD analysis performed on both primary and metastatic tumor specimens. For these patients, the HRD score was significantly higher in the metastasis versus the primary tumor biopsy (mean difference in pairs of 9.4 ± 8.1, *P* = 0.01 by paired *t*-test). Thus, HRD scores are readily obtainable from archival FFPE specimens and metastatic biopsies may yield higher HRD scores compared to the primary tumor.

Next, we assessed whether HRD scores could serve as a biomarker of response to talazoparib therapy. The continuous treatment response by change in tumor size as measured by RECIST criteria (sum of longest diameters, SLD) was plotted as a function of the tumor HRD score (Fig. 2b). In cases where more than one HRD score was available per patient, the higher score was used. This analysis yielded a positive correlation between continuous treatment response and HRD score, with higher HRD scores associated with better response to therapy (Pearson’s *r* = 0.64, *P* = 0.008). In particular, all five assayed tumors derived from patients with g*PALB2* (one g*PALB2* tumor HRD score failed) passed the HRD cutoff of 33, and four out of five passed the HRD cutoff of 42. The HRD threshold of ≥33 captures 99% of known *BRCA1*- and/or *BRCA2*-deficient ovarian cancers, whereas the HRD score of ≥42 captures 95% of known *BRCA1*- and/or *BRCA2*-deficient ovarian cancers^27^, but cutoffs are less well defined for other tumor types. This correlation was primarily driven by the patients with g*PALB2* mutations as removal of these patients from the analysis yielded a nonsignificant result (*r* = 0.03, *P* = 0.92 by Pearson’s correlation). This indicates that HRD score may capture patients in addition to those with *gBRCA1*/*2* mutations who benefit from PARP inhibitor therapy. Namely, patients with g*PALB2* mutations have tumors with a high degree of genomic instability that mirror g*BRCA1*/*2-*mutated tumors.

### Tumor sequencing and LOH

**Fig. 3:**
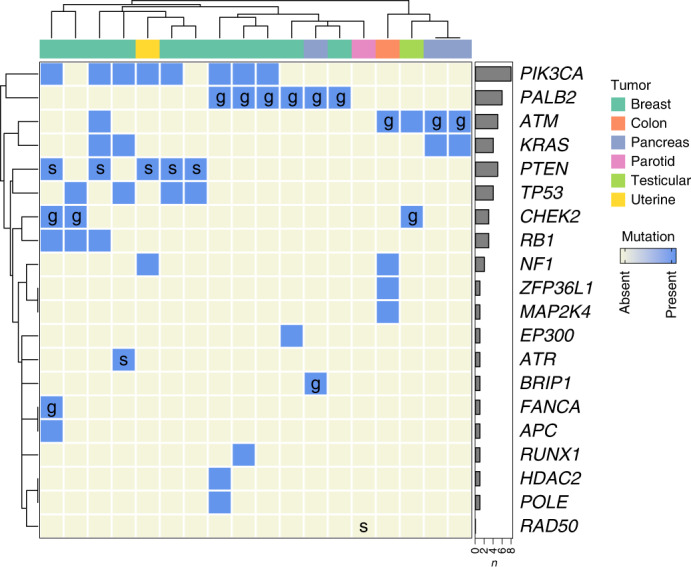
Somatic mutations and LOH identified by NGS. Heatmap of next-generation panel sequencing (108 genes interrogated) for HRD-associated and cancer-associated mutations in tumor specimens from patients treated in cohort B (*n* = 18 patients, 2 excluded due to insufficient sample). Germline (g) and somatic (s) mutations used for study enrollment are indicated. Hierarchical clustering is by Euclidean distance. Bar graphs depict numbers of mutations of each type detected across the cohort. Tumor type is indicated by color. See Supplementary Table 3 for a list of genes assayed. Source data

As genomic instability was positively correlated with continuous treatment response to talazoparib, we performed a deeper interrogation of genomic mutations in these tumors. Primary and metastatic samples were sequenced with a hybridization capture panel of 108 genes associated with HRD in human cancers (see gene list in Supplementary Table 3). Genomic mutations in primary and metastatic lesions were binned. The data are presented as a heatmap (Fig. 3). The most common alterations detected included mutations in *PIK3CA* (*n* = 8), *PALB2* (*n* = 6), *ATM* (*n* = 5), *KRAS* (*n* = 4), *PTEN* (*n* = 5) and *TP53* (*n* = 4). In all cases except one, the HR-associated mutation detected by CLIA-approved NGS used as an entry criterion was detected (s*RAD50* in the parotid tumor was not detected). This indicates that these alterations are likely to be present in a high allelic fraction of the sampled tumors and therefore likely contribute to either disease onset or malignant progression.

This NGS panel assay allowed us to explore LOH at the assayed genes (Supplementary Table 4). Of the tumors with g*PALB2* mutations, three of the six tumors had LOH for *PALB2*, and an additional two tumors had two independent *PALB2* mutations, suggesting biallelic inactivation, although further studies would be needed to confirm this. Other detected mutations that were associated with LOH included all s*TP53* mutations (*n* = 4), all g*CHEK2* mutations (*n* = 3), a g*FANCA* mutation (*n* = 1), all s*RB1* mutations (*n* = 3) and an *NF1* mutation (*n* = 1). Of the three tumors with g*ATM* mutations, one had LOH while the others (*n* = 2) had two independent mutations. s*ATM* mutations were associated with LOH in a breast cancer sample and with two independent mutations in testicular cancer. Tumors with evidence of LOH had higher HRD scores (means of 39 ± 19 versus 22 ± 16, respectively), although statistical significance was not reached (*p* = 0.07 by Welch’s). Thus, multiple genes associated with HRD are likely associated with LOH in tumors, especially in those with g*PALB2*, g*CHEK2* and g*ATM*/s*ATM* mutations.

### Circulating tumor DNA sequencing at baseline and progression

**Fig. 4:**
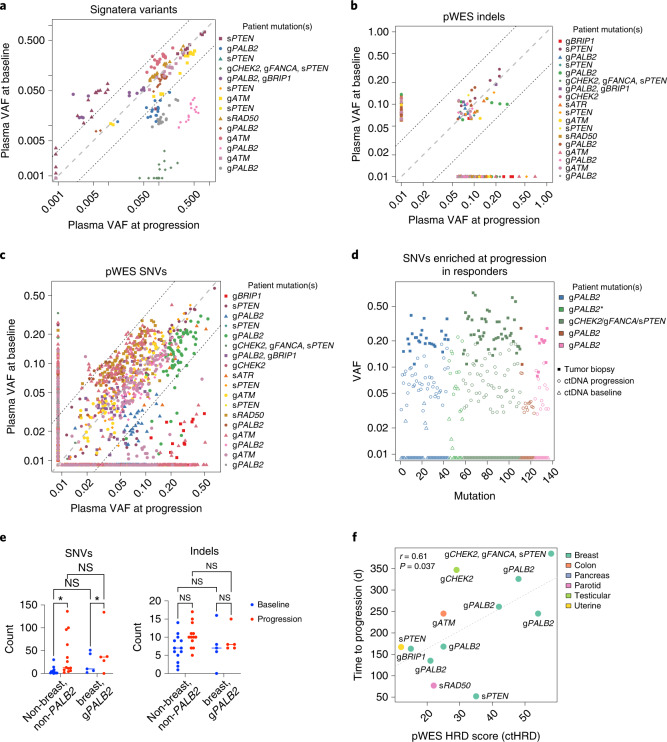
ctDNA variants and ctHRD score correlating with treatment outcomes. **a**, Variants detected by Signatera assay of baseline and progression ctDNA samples plotted by VAF. Dashed lines represent fivefold difference cutoffs, *n* = 14 patients. **b**, pWES-identified indels (*n* = 18 patients) at baseline and progression. **c**, SNVs identified by pWES at baseline and progression (*n* = 18 patients). **d**, Deleterious SNVs enriched fivefold in progression samples (*n* = 6 patients) from responders (decrease of >20% in SLD) compared to baseline. Variants identified in tumor WES are shown (an anterisk indicates insufficient sample for tumor WES). **e**, ctDNA SNV counts are significantly increased in progression samples compared to control samples (*n* = 18 patients), whereas there is no significant difference in indel counts (two-way ANOVA with Tukey’s correction, *P = 0.033; median indicated by horizontal line). NS, not significant. **f**, scarHRD was used to calculate ctHRD score from baseline pWES and correlated with time to progression in days. The correlation coefficient (*r*) and two-sided *P* value were calculated with Pearson’s method and are depicted as a dashed line. *n* = 12 patients. Source data

**Extended Data Fig. 2:**
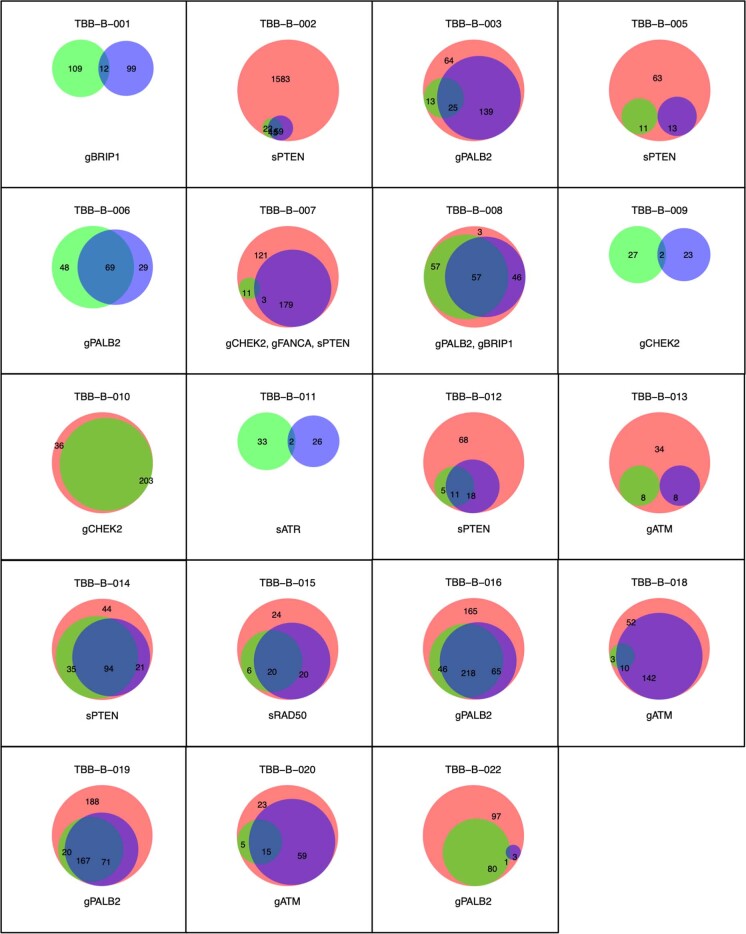
Overlap of variant detection by tumor WES and plasma WES. Number of detected variants by tumor WES (red), plasma WES at baseline (green) and plasma WES at progression (blue) depicted as proportional venn diagrams. Patient study number and qualifying genetic mutation are above and below, respectively. Source data

Given that we observed characteristic LOH associated with germline mutation of DNA repair genes, we pursued further genomic characterization of patient materials. Plasma was prospectively collected from patients at study enrollment (baseline) and at the time of disease progression for exploratory studies of cell-free DNA (cfDNA). Circulating tumor DNA (ctDNA) from plasma was sequenced with two technologies: (1) the commercial Signatera assay and (2) plasma whole-exome sequencing (pWES), which was performed independently of tumor biopsy sequencing, but was compared to normal tissue to isolate tumor-associated mutations (Fig. 4a–c). The variant allele fraction (VAF) for each single-nucleotide variant (SNV) or indel is plotted at baseline and progression. Of the 19 patient samples available for tumor WES, 4 were excluded due to hypocellularity and/or low genomic DNA recovery. The remaining 15 patients had successful design of 16-plex Signatera assays. Signatera-based ctDNA was detected in 25 of 29 plasma samples (86%; Fig. 4a). pWES detected 1,493 and 1,771 SNVs in ctDNA at baseline and progression, respectively (Fig. 4c), compared to 3,464 variants detected in tumor tissue. There was no significant difference in the number of overlapping mutations per sample between baseline ctDNA and tumor tissue compared to progression ctDNA and tumor tissue (13 ± 33 and 38 ± 75 (median ± interquantile range (IQR)), respectively; *P* = 0.16 by Wilcoxon signed-rank test; Extended Data Fig. 2). This indicates that pWES of ctDNA can detect tumor-associated SNVs and indels in an unbiased manner, which partially, but not completely, overlap with variants detected by tumor sequencing.

**Extended Data Fig. 3:**
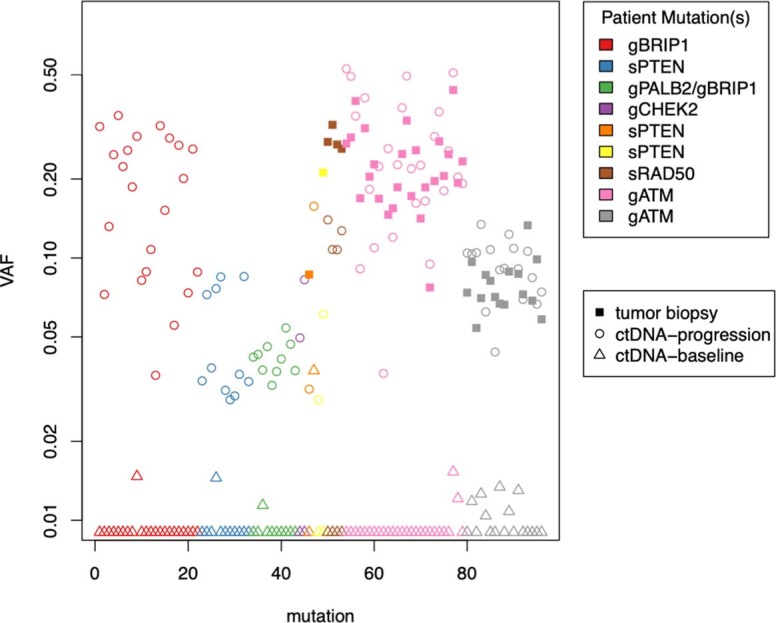
Deleterious ctDNA SNVs enriched at progression in non-responders. Deleterious ctDNA SNVs enriched 5-fold in progression samples from non-responders (change of SLD > -20%) compared to baseline. Deleterious variants identified in tumor WES are also shown. N = 9 patients. Source data

Unbiased detection of ctDNA variants at baseline and progression allowed us to query whether certain variants were associated with talazoparib response or resistance. There were 136 deleterious variants that increased in frequency by more than fivefold in progression samples compared to baseline samples in responders (patients who had evidence of tumor shrinkage of at least 20% from baseline; Fig. 4d). Although indels resulting in the reversion of initiating HRD mutations have been previously reported as a mechanism of resistance to PARP inhibitors^28,29^, we did not detect any such mutations in this dataset, despite mean coverages of 600× and 450× across the exome panel and the *PALB2* gene, respectively. A significant increase in SNVs, but not indels, was detected in both responders (patients with breast cancer with g*PALB2* mutations) and other patients at progression compared to baseline (Fig. 4e and Extended Data Fig. 3). Taken together, these results suggest that specific mutations associated with therapy resistance were potentially identified in our dataset, although further confirmation of their relevance to cancer progression is needed.

Although specific mutations could be associated with resistance to PARP inhibitor therapy, we also sought to identify additional biomarkers of response to therapy. Our previous results indicated that HRD score based on tumor sequencing correlated with continuous response to talazoparib as measured by a change in SLD (Fig. 2b). Thus, an HRD score derived from pWES of ctDNA (here termed ctHRD) was also assessed as a biomarker of PARP inhibitor response. Recently, a computational workflow (scarHRD) has been established to calculate HRD scores from tumor NGS data^30^. This algorithm was applied in the tumor and ctDNA WES datasets. The scarHRD score calculated from tumor WES was highly correlated to the Myriad HRD score on tumor-derived samples (*r* = 0.85, *P* = 0.004 by Pearson’s method) indicating that the scarHRD metric may be a suitable proxy for the Myriad HRD test.

Next, we assessed the relationship between ctHRD levels and treatment response. When focused on the patients with abundant detected ctDNA (Signatera VAF > 20%, *n* = 12), a significant correlation was observed between baseline ctHRD score and continuous time to progression after talazoparib treatment (Fig. 4f, *r* = 0.61, *P* = 0.037, by Pearson’s method), but correlations with tumor shrinkage were not found to be statistically significant. In contrast, ctHRD scores calculated from progression time points were not significantly associated with time to progression (*r* = 0.38, *P* = 0.22, by Pearson’s method). This could not be accounted for by a decrease in progression VAF, as progression samples had a slight but significant increase in VAF compared to baseline (5.7% ± 10.5% versus 4.9% ± 10.4% (median ± IQR) for progression versus baseline). This indicates that baseline measurement of ctHRD could be considered as a potential biomarker of benefit from PARP inhibitor therapy and that ctHRD scores may evolve during cancer progression or therapy.

### Evaluation of tumor mutational signatures

**Fig. 5:**
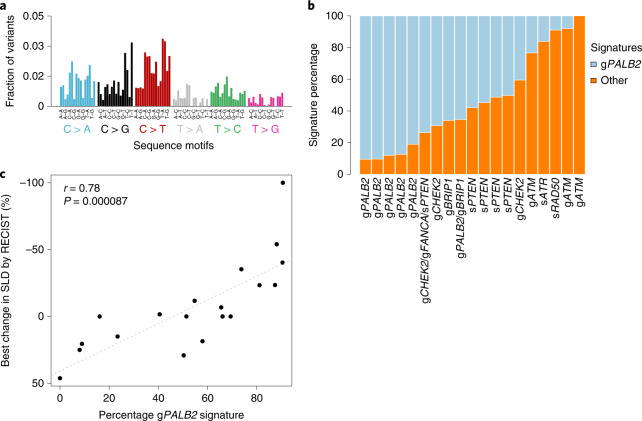
Mutational signature analysis and correlation with treatment outcome. **a**, De novo mutational signatures were computed using all available mutation data including tumor exome and plasma ctDNA exome sequencing. The predominant signature detected in the g*PALB2* samples is shown. *n* = 19 patients. **b**, Mutational signatures were binned by whether they included the g*PALB2*-associated signature (HRD) or other signatures and plotted as a percentage of total SNVs. *n* = 19 patients. **c**, Treatment response to talazoparib (best change in SLD by RECIST) is plotted according to percentage contribution of HRD signature derived from tumor exome and plasma ctDNA sequencing. Correlation by Pearson’s method is depicted by the dashed line, with the two-sided *P* value. *n* = 19 patients. Source data

Although our results indicate that HRD and ctHRD scores could be biomarkers of talazoparib response, other indices of HRD have also been suggested, including tumor mutational signatures^31,32^. We performed an exploratory de novo mutational signature analysis using all collected variants from tumor and ctDNA sequencing for all patients. One signature was predominant in the samples collected from patients with only g*PALB2* mutations, making up >80% of the mutational signatures detected in these samples (Fig. 5a,b). This signature strongly resembled Catalog of Somatic Mutations in Cancer (COSMIC) signature 3 that has a proposed etiology of defective HR DNA damage repair^32^. This signature was assessed as a biomarker of talazoparib treatment response. A positive correlation (*r* = 0.78, *P* = 8.7 × 10^−5^ by Pearson’s method) was detected between the percentage of the g*PALB2* signature and the best change in SLD by RECIST (Fig. 5c). When only the tumor WES results were used for mutational signature detection, the positive correlation between the g*PALB2* signature and treatment response remained (*r* = 0.79, *P* = 0.0052 by Pearson’s method). Additionally, these correlations were not driven primarily by the g*PALB2* samples because when these samples were withheld, the correlation remained (*r* = 0.74, *P* = 0.0091 by Pearson’s method). Therefore, the g*PALB2* samples had a defining mutational signature that resembled a known HRD-associated signature, and the presence of this signature correlated with PARP inhibitor response across all samples.

## Discussion

This single-agent phase II study was designed to determine whether genomic biomarkers other than g*BRCA1*/*2* mutations could be used to select patients for PARP inhibitor monotherapy in the advanced or metastatic setting. Cohort B of the Talazoparib Beyond BRCA trial was specifically assessed by either germline or somatic mutations in a panel of genes associated with HR pathway activity as entry criteria. Our results demonstrate that this may be a useful strategy especially in patients with metastatic HER2-negative breast cancer in whom we report a 31% ORR. These positive results were driven in large part by individuals with g*PALB2* mutations, as all five breast cancers with this entry criterion (and one pancreatic cancer patient with g*PALB2* and g*BRIP1* mutations) had tumor shrinkage as the best response. Our results were first reported at the American Society of Clinical Oncology (ASCO) 2019 (ref. ^33^) and then subsequently corroborated in the recently reported TBCRC 048 Olaparib Expanded study reported at ASCO 2020 (ref. ^34^). In our study, tumors with g*PALB2* mutations had uniformly high HRD scores, which led to a positive correlation in our cohort between high tumor HRD scores and magnitude of tumor response to talazoparib monotherapy in this setting. In contrast, tumors with other mutations including g*ATM* and g*CHEK2* mutations had LOH, but not increased HRD scores, as predicted by previous laboratory studies^35,36^. One responding patient had combined mutations including g*FANCA*, g*CHEK2* and s*PTEN* mutations (with LOH for *FANCA*), perhaps highlighting the role of *FANCA* in PARP inhibitor sensitivity^10,11,37,38^. In the TBCRC 048 study, 9 of 11 patients with a g*PALB2* mutation responded, although further genomic analyses are pending.

The *PALB2* gene product forms a stable biochemical complex with BRCA2 protein and functions in HR-mediated gene repair by recruiting and stimulating the strand invasion activity of RAD51, to overcome replication protein A (RPA) binding to ssDNA^39–44^. Previous studies have identified that it has a scaffolding function in nucleating a BRCA1–BRCA2 complex, stabilizes BRCA2 protein levels and is required for HR-mediated DNA repair^39,41,45^. Given this strong scientific rationale, it is perhaps unsurprising that g*PALB2* mutations appear to closely phenocopy g*BRCA1*/*2* mutations in conferring sensitivity to PARP inhibition in our study. The functional relevance of g*PALB2* in HR-mediated DNA repair is buttressed by our finding of high HRD scores in tumors from these individuals. In addition, other studies have identified tumors with g*PALB2* mutations as sensitive to PARP inhibitor therapy^11,34,46,47^.

PARP inhibitors represent an orally available, well-tolerated, single-agent therapy. The identification of further biomarkers to expand access to this type of treatment to tumors beyond g*BRCA1*/*2*-mutated advanced breast or ovarian cancers would have a meaningful impact for patients otherwise treated with infusional chemotherapies. Our study suggests that breast or pancreatic cancers with g*PALB2* mutations should be pursued more extensively in larger clinical trials for PARP inhibitor monotherapy. In breast cancer, g*PALB2* mutations confer a higher risk of various breast cancer subtypes including hormone receptor-positive, HER2-positive and triple-negative breast cancer, in proportions similar to those seen with g*BRCA2* mutations^48^. Individuals with g*PALB2* mutations have increased risks for breast cancer (odds ratio (OR) of ~3.8–5.0), male breast cancer (OR of ~10) and pancreatic cancer (OR of ~1.5–2.0)^49^. The elevated risk for ovarian cancer is modest and influenced by familial factors, whereas there are no consistent elevated risks reported for prostate or colorectal cancers^49^. In our study, the g*PALB2-*associated tumors tended to have LOH and high HRD scores. The HRD assay could be considered as a qualifying biomarker for PARP inhibitor therapy, although further studies are required to determine whether HRD scores independently predict treatment responses beyond targeted mutation sequencing for g*BRCA1*/*2* or g*PALB2*. Additionally, our finding that metastatic biopsies had higher HRD scores than primary tumors may reflect ongoing accumulation of genomic scars in HR-deficient tumors^50^. This could suggest a preference of metastatic biopsies for HRD scores in future clinical trials. The use of an HRD score to select patients for PARP inhibitor therapy is currently under study in cohort A of the Talazoparib Beyond BRCA study, specifically in patients with advanced or metastatic triple-negative breast cancer, although this design could also be contemplated for other tumor types.

Genomic analyses including tumor panel and exome sequencing as well as WES of plasma ctDNA have allowed us to explore additional potential biomarkers of talazoparib response. Among potential biomarkers, we identify a tumor mutational signature prevalent in the g*PALB2* samples and present to a lesser extent in other tumors that correlated with tumor response. This mutational signature strongly resembled the known HRD-associated signature 3, which has been previously attributed to tumors bearing g*BRCA1*/*2* mutations and was also detected in the g*PALB2* samples previously^51^. Additionally, we calculated ctHRD scores from baseline plasma samples and found this score to be a potential biomarker for duration of treatment response. We also note that ctHRD scores evolve with therapy and could be further investigated as a noninvasive, dynamic indicator of sensitivity to PARP inhibitor therapy.

In summary, the Talazoparib Beyond BRCA trial is a prospective study that identifies the sensitivity of g*PALB2* breast cancers to PARP inhibition and highlights the core role of PALB2 in BRCA1- and/or BRCA2-mediated HR DNA repair in human breast cancers. These results are currently being further evaluated in a multi-institutional study, ‘Talazoparib monotherapy in *PALB2* mutation associated advanced breast cancer’ (NCT04756765). These efforts may confirm a patient population that benefits from targeted therapy to improve patient outcomes and diminish toxicity associated with chemotherapies that are commonly used for these patients.

## Methods

This research was approved by the Stanford University Institutional Review Board (IRB-31913), and patients were treated at the Stanford Cancer Center. The clinical trial was performed in accordance with the International Ethical Guidelines for Biomedical Research Involving Human Subjects, the International Conference on Harmonization Guidelines for Good Clinical Practice and the Declaration of Helsinki. All patients provided written informed consent before enrollment. Participants did not receive compensation.

### Trial design, patient selection and treatment

Talazoparib Beyond BRCA (NCT02401347, registered 27 March 2015) is a phase II, open-label, non-randomized single-institution trial that enrolled patients in two separate cohorts using an optimal two-stage design. Cohort A is ongoing and is enrolling patients with pretreated advanced triple-negative breast cancer with an elevated HRD score (≥42 using Myriad MyChoice HRD CDx). Cohort B enrolled patients (August 2015 to December 2018) with HER2-negative breast cancer or another non-breast advanced solid tumor associated with a germline or somatic pathogenic variant in select HR DNA repair pathway genes excluding *BRCA1* and *BRCA2* (*PALB2*, *CHEK2*, *ATM*, *NBN*, *BARD1*, *BRIP1*, *PTEN*, *MRE11*, *ATR*, *RAD50*, *RAD51C*, *RAD51D*, *FANCA*, *FANCC*, *FANCD2*, *FANCE*, *FANCF*, *FANCG*, *FANCL*). Eligible patients for cohort B had histologically confirmed metastatic or recurrent HER2-negative breast cancer or another metastatic solid tumor and measurable disease per RECIST 1.1 (ref. ^52^). Patients were required to have experienced previous progression of disease on at least one line of therapy for metastatic or unresectable locally advanced disease, and there was no upper limit on the number of previous therapies. Eligible patients had a known deleterious or suspected deleterious pathologic variant identified by a CLIA-approved NGS tumor or germline assay. Patients were excluded if they harbored a deleterious germline or somatic pathologic variant in *BRCA1* or *BRCA2* and if they had had a history of previous disease progression on platinum-containing therapy or within 8 weeks of the last platinum dose. Patients were treated with talazoparib 1 mg orally daily on a continuous schedule with dose reductions permissible in line with the FDA label. Therapy was continued until there is disease progression or unacceptable toxicity. Restaging scans were performed every 8 weeks until cycle 8 at which point scans could occur every 12 weeks. The study protocol was updated to allow for enrollment of non-breast cancer solid tumors.

### Objectives and endpoints

The primary objective was to determine whether single-agent talazoparib can result in a 30% or greater rate of objective response. Secondary objectives included determination of the CBR, PFS and safety. Correlative objectives included comparison of HRD scores in responders versus non-responders and assessment of the concordance of HRD scores in primary versus metastatic tumors. Plasma was prospectively collected at baseline and after progression for exploratory cfDNA assessment. Objective tumor treatment responses were scored by RECIST 1.1 criteria. CBR was defined as CR, PR or SD at ≥ 24 weeks per RECIST v1.1. PFS was evaluated using the Kaplan–Meier method. To assess safety of talazoparib in this study population, adverse events were graded using CTCAE v5 and summarized descriptively.

### Statistics and reproducibility

A two-stage design was used for the enrollment of study participants separately in cohort A and cohort B with a set null hypothesis of ≤ 5% ORR and an alternative response rate of ≥ 30%. Interim analyses were to be performed, separately in each cohort, after accrual of ten response-assessable patients. If at least 2 of the 10 patients responded, then 10 additional patients were to be enrolled for a total of 20 patients in each cohort. Based on our statistical constraints, at least 3 patients of the 20 patients must respond in each cohort to declare statistical significance at a one-sided 5% level with 80% power or better. No data were excluded from the prespecified analyses. There was no treatment randomization; investigators were not blinded to outcome assessments. Further information on research design is available in the Nature Research Reporting Summary linked to this article.

### Correlative studies

Tumors with g*BRCA1*/*2* mutations demonstrate HRD, which can be quantified by the NGS metrics of LOH, telomeric allelic imbalance and large-scale state transitions. Summation of these metrics leads to a combined HRD score (range, 0–100) with higher values indicative of a higher burden of genomic alterations due to HRD, which has been referred to as a ‘genomic scar’ (ref. ^53^). HRD scores were assessed on FFPE tumor tissues by myChoice HRD CDx assay (Myriad^54^). Metastatic biopsy samples were requested, but primary tumor samples were allowed if additional metastatic biopsy was contraindicated or infeasible. NGS of FFPE tumor tissue was performed using a 108-gene panel assay (Myriad). The Signatera assay^55^ and ctDNA WES and tumor WES were performed by Natera. WES of the tumor was performed on germline DNA isolated from ten FFPE slides or tissue blocks. Matched normal WES was performed from germline DNA isolated from 1 ml of buffy coat. Signatera was performed according to a standard workflow including design of up to 16-plex patient-specific somatic assays based on tumor–normal WES. Plasma samples from EDTA BCT tubes were processed for cfDNA extraction, library preparation, bespoke multiplex PCR with appropriate Signatera assays, NGS (HiSeq 2500) and analysis. SNP genotype concordance between tissue and normal samples for WES and between normal and plasma samples was verified. Tumor tissue was sequenced to a mean deduplicated coverage of 180× with uniformity of >70% of target bases having >100× deduplicated coverage. Matched normal WES was performed to a mean deduplicated coverage of 50× with a uniformity of >70% of target bases achieving >30× deduplicated coverage. Median-extracted cfDNA metrics were as follows: 16.7 ng ml^-1^ of plasma and 44.7 ng total of cfDNA across all 29 plasma samples from 15 patients. WES of plasma ctDNA was performed using a portion of the Signatura libraries. Sequencing was carried out on the Illumina Novaseq platform at >200× coverage. Somatic variant calling of pWES was performed by state-of-the-art variant callers accompanied by proprietary filtering approaches. Variants previously reported to be germline in public datasets such as dbSNP and population studies were also filtered out to avoid germline variant selection. Tumor variants were visualized by ComplexHeatmap^56^. ctHRD was calculated by first deriving allele-specific copy number profiles from pWES with Sequenza^57^ followed by scarHRD^30^. Proportional Venn diagrams were created with the BioVenn R package^58^. De novo mutational signatures were calculated with the mutSignatures R package^59^. ggPlot2 (ref. ^60^) was used to generate graphics. All statistical tests are two-sided unless otherwise stated. Data distribution was assumed to be normal, but this was not formally tested. Data collection and analysis were not performed with blinding to the conditions of the experiments. Data points were not excluded from analysis.

### Reporting summary

Further information on research design is available in the Nature Research Reporting Summary linked to this article.

## Supplementary information

Supplementary Information Supplementary Tables 1–4.

Reporting Summary

## Data Availability

The individual tumor genomic data including raw NGS data and individualized clinical annotations have been deposited at dbGaP (accession number phs002803). The pWES data were used under license for the current study and so are not publicly available, but these may be provided by Natera on reasonable request. Summarized clinical data, sequencing results source data and the original clinical trial protocol are provided as Supplementary Information. All other data supporting the findings of this study are available from the corresponding author on reasonable request. Source data are provided with this paper.

## Source data

Source Data Fig. 1 Statistical source data.
Source Data Fig. 2 Statistical source data.
Source Data Fig. 3 Statistical source data.
Source Data Fig. 4 Statistical source data.
Source Data Fig. 5 Statistical source data.
Source Data Extended Data Fig. 2 Statistical source data.
Source Data Extended Data Fig. 3 Statistical source data.

## Code availability

Custom R scripts used to analyze the data, along with input data, are available at https://github.com/GruberLabUTSW/TBB.
