# Supplementary material for: A phase II study of talazoparib monotherapy in patients with wild-type BRCA1 and BRCA2 with a mutation in other homologous recombination genes
Source: Nat Cancer. 2022 Oct 17;3(10):1181–91. doi: 10.1038/s43018-022-00439-1 (PMC9586861; doi:10.1038/s43018-022-00439-1)
Supplement: Supplementary file 1 — Supplementary Tables 1–4. [file 43018_2022_439_MOESM1_ESM.pdf]

nature cancer

Article https://doi.org/10.1038/s43018-022-00439-1

A phase II study of talazoparib monotherapy in patients with wild-type *BRCA1* and *BRCA2* with a mutation in other homologous recombination genes

In the format provided by the authors and unedited

Pre-specified cohort B mutations	
PALB2	RAD51C
CHEK2	RAD51D
ATM	FANCA
NBN	FANCC
BARD1	FANCD2
BRIP1	FANCE
PTEN	FANCF
MRE11	FANCG
ATR	FANCL
RAD50	

Supplementary Table 1: Pre-specified cohort B mutations. Protocol-specified germline or somatic mutations associated with HR-deficiency eligible for enrollment to cohort B. Other mutations were considered based on Investigator assessment of likelihood of mutation to impair HR.

Hematologic			
	**All Grade % (n)**	**Grade 3 % (n)**	**Grade 4 % (n)**
Number of patients ≥ 1 AE	55 (11)	30 (6)	0
Anemia	20 (4)	15 (3)	0
Thrombocytopenia	45 (9)	15 (3)	0
Neutropenia	30 (6)	10 (2)	0
**Non-Hematologic**			
	**All Grade % (n)**	**Grade 3 % (n)**	**Grade 4 % (n)**
Number of patients ≥ 1 AE	70 (14)	5 (1)	0
Gastrointestinal	65 (13)	0	0
Nausea	45 (9)	0	0
Constipation	15 (3)	0	0
Vomiting	10 (2)	0	0
Diarrhea	10 (2)	0	0
Anorexia	10 (2)	0	0
AST/ALT increased	5 (1)	0	0
Mucositis oral	5 (1)	0	0
Fatigue	20 (8)	5 (1)	0
Alopecia	10 (2)	0	0
Headache	5 (1)	0	0

Supplementary Table 2: Treatment-related adverse events. Subset of adverse-events (AE) related to talazoparib treatment by investigator assessment for all (n=20) patients enrolled in cohort B, separated by hematologic and non-hematologic. All treatment-related AEs of grade ≥ 3 are included here. n= number of patients.

Genes interrogated by next-generation sequencing			
AKT1	ELF3	KMT2B	PSMC3IP
APC	EMSY	KMT2D	PTEN
AR	EP300	KRAS	PTPRD
ARID1A	EPCAM	MAP2K1	RAD51
ATM	ERBB2	MAP2K2	RAD51B
ATR	ERCC1	MAP2K4	RAD51C
AXIN2	ERCC3	MAP3K1	RAD51D
BARD1	FAM175A	MET	RAD54L
BLM	FANCA	MLH1	RB1
BMPR1A	FANCI	MLH3	RECQL
BRAF	FANCL	MRE11A	RET
BRCA1	FANCM	MSH2	RHOA
BRCA2	FBXW7	MSH3	RHOB
BRIP1	FGFR1	MSH6	RINT1
BTG2	FGFR2	MYH	RPS20
CCND3	FGFR3	NBN	RUNX1
CCNE1	FGFR4	NF1	RXRA
CDH1	FOXA1	NFE2L2	SF3B1
CDK12	FOXQ1	NRAS	SLX4
CDK4	GALNT12	NTHL1	SMAD4
CDKN2A	GATA3	PAIP1	STAG2
CHEK1	GREM1	PALB2	STK11
CHEK2	HDAC2	PIK3CA	TOP1
CSMD3	HOXB13	PMS2	TP53
CTCF	KDM6A	POLD1	TSC1
DDR2	KIT	POLE	TXNIP
EGFR	KLF5	PPP2R2A	ZFP36L1

Supplementary Table 3: Genes assayed by next-generation sequencing of tumor specimens for HRD- and cancer-associated gene mutations.

Mutation	n	LOH detected	Secondary mutations	Other
gATM	3	1	2	
gBRIP1	2			i, u
gCHEK2	3	3		
gFANCA	1	1		
gPALB2	6	3	2	i
sATM	2	1	1	
sATR	1	0		
sPTEN	5	1	2	u, f
sRAD50	1			nd
sTP53	4	4		
sRB1	3	3		

Supplementary Table 4: LOH analysis of mutations used for entry criteria. n= number of mutations detected. Secondary mutations included either deleterious SNV, frameshift mutation or large-scale rearrangement. i = insufficient sample, u = uncertain, f = failed, nd = not detected on follow-up tumor sequencing.
