# Supplementary material for: A phase II study of talazoparib monotherapy in patients with wild-type BRCA1 and BRCA2 with a mutation in other homologous recombination genes
Source: Nat Cancer. 2022 Oct 17;3(10):1181–91. doi: 10.1038/s43018-022-00439-1 (PMC9586861; doi:10.1038/s43018-022-00439-1)
Supplement: Supplementary file 2 — Reporting Summary [file 43018_2022_439_MOESM2_ESM.pdf]

nature portfolio

Corresponding author(s): Joshua Gruber
Melinda Telli

Last updated by author(s): 7/30/2022

Reporting Summary

Nature Portfolio wishes to improve the reproducibility of the work that we publish. This form provides structure for consistency and transparency in reporting. For further information on Nature Portfolio policies, see our Editorial Policies and the Editorial Policy Checklist.

Statistics

For all statistical analyses, confirm that the following items are present in the figure legend, table legend, main text, or Methods section.

n/a	Confirmed	
☐	☒	The exact sample size (*n*) for each experimental group/condition, given as a discrete number and unit of measurement
☐	☒	A statement on whether measurements were taken from distinct samples or whether the same sample was measured repeatedly
☐	☒	The statistical test(s) used AND whether they are one- or two-sided<br>*Only common tests should be described solely by name; describe more complex techniques in the Methods section.*
☐	☒	A description of all covariates tested
☐	☒	A description of any assumptions or corrections, such as tests of normality and adjustment for multiple comparisons
☐	☒	A full description of the statistical parameters including central tendency (e.g. means) or other basic estimates (e.g. regression coefficient) AND variation (e.g. standard deviation) or associated estimates of uncertainty (e.g. confidence intervals)
☐	☒	For null hypothesis testing, the test statistic (e.g. *F*, *t*, *r*) with confidence intervals, effect sizes, degrees of freedom and *P* value noted<br>*Give P values as exact values whenever suitable.*
☒	☐	For Bayesian analysis, information on the choice of priors and Markov chain Monte Carlo settings
☐	☒	For hierarchical and complex designs, identification of the appropriate level for tests and full reporting of outcomes
☐	☒	Estimates of effect sizes (e.g. Cohen's *d*, Pearson's *r*), indicating how they were calculated

Our web collection on statistics for biologists contains articles on many of the points above.

Software and code

Policy information about availability of computer code

Data collection	No software was used for data collection.

Data analysis

```
R version 3.6.1 (2019-07-05)
Platform: x86_64-apple-darwin15.6.0 (64-bit)
Running under: macOS  10.16

Matrix products: default
LAPACK: /Library/Frameworks/R.framework/Versions/3.6/Resources/lib/libRlapack.dylib

Random number generation:
 RNG:     Mersenne-Twister
 Normal:  Inversion
 Sample:  Rounding

locale:
[1] en_US.UTF-8/en_US.UTF-8/en_US.UTF-8/C/en_US.UTF-8/en_US.UTF-8

attached base packages from R v 3.6.1:
 [1] stats4    parallel  grid      stats     graphics  grDevices utils     datasets  methods   base

other attached packages:
```

```
 [1] mutSignatures_2.1.1          foreach_1.5.1               BSgenome.Hsapiens.UCSC.hg19_1.4.0
 [4] BSgenome_1.54.0              rtracklayer_1.46.0          Biostrings_2.54.0
 [7] XVector_0.26.0               GenomicRanges_1.38.0        GenomeInfoDb_1.22.1
[10] IRanges_2.20.2               S4Vectors_0.24.4            BiocGenerics_0.32.0
[13] gridExtra_2.3                ggplot2_3.3.0               reshape2_1.4.4
[16] dplyr_1.0.2                  BioVenn_1.1.3               tidyr_1.0.3
[19] scales_1.1.1                 stringr_1.4.0               circlize_0.4.9
[22] ComplexHeatmap_2.5.3         devtools_2.3.1              usethis_1.6.1
[25] RColorBrewer_1.1-2

loaded via a namespace (and not attached):
 [1] matrixStats_0.56.0      bitops_1.0-6            fs_1.4.1                bit64_0.9-7
 [5] doParallel_1.0.16       progress_1.2.2          httr_1.4.1              rprojroot_1.3-2
 [9] tools_3.6.1             backports_1.1.7         R6_2.4.1                DBI_1.1.0
[13] colorspace_1.4-1        GetoptLong_0.1.8        withr_2.2.0             tidyselect_1.1.0
[17] prettyunits_1.1.1       processx_3.4.2          bit_1.1-15.2            curl_4.3
[21] compiler_3.6.1          cli_2.0.2               Biobase_2.46.0          DelayedArray_0.12.3
[25] desc_1.2.0              labeling_0.3            proxy_0.4-26            callr_3.4.3
[29] askpass_1.1             rappdirs_0.3.1          Rsamtools_2.2.3         systemfonts_1.0.2
[33] digest_0.6.25           svglite_2.0.0           pkgconfig_2.0.3         sessioninfo_1.1.1
[37] plotrix_3.7-8           dbplyr_1.4.3            rlang_0.4.11            GlobalOptions_0.1.1
[41] rstudioapi_0.11         RSQLite_2.2.0           farver_2.0.3            shape_1.4.4
[45] generics_0.0.2          BiocParallel_1.20.1     RCurl_1.98-1.2          magrittr_1.5
[49] GenomeInfoDbData_1.2.2  Matrix_1.2-18           Rcpp_1.0.5              munsell_0.5.0
[53] fansi_0.4.1             lifecycle_0.2.0         stringi_1.4.6           SummarizedExperiment_1.16.1
[57] zlibbioc_1.32.0         pkgbuild_1.0.8          plyr_1.8.6              BiocFileCache_1.10.2
[61] blob_1.2.1              crayon_1.3.4            lattice_0.20-41         hms_0.5.3
[65] ps_1.3.3                pillar_1.4.6            rjson_0.2.20            codetools_0.2-16
[69] biomaRt_2.42.1          pkgload_1.0.2           XML_3.99-0.3            glue_1.4.2
[73] remotes_2.2.0           BiocManager_1.30.10     png_0.1-7               vctrs_0.3.4
[77] testthat_2.3.2          gtable_0.3.0            openssl_1.4.1           purrr_0.3.4
[81] clue_0.3-57             assertthat_0.2.1        pracma_2.3.3            tibble_3.0.3
[85] iterators_1.0.13        GenomicAlignments_1.22.1 AnnotationDbi_1.48.0   memoise_1.1.0
[89] cluster_2.1.0           ellipsis_0.3.1
```

For manuscripts utilizing custom algorithms or software that are central to the research but not yet described in published literature, software must be made available to editors and reviewers. We strongly encourage code deposition in a community repository (e.g. GitHub). See the Nature Portfolio guidelines for submitting code & software for further information.

Data

Policy information about availability of data

All manuscripts must include a data availability statement. This statement should provide the following information, where applicable:

- Accession codes, unique identifiers, or web links for publicly available datasets
- A description of any restrictions on data availability
- For clinical datasets or third party data, please ensure that the statement adheres to our policy

The individual tumor genomic data including raw next-generation sequencing data and individualized clinical annotations have been deposited at dbGAP (access number phs002803). The plasma WES data were used under license for the current study, and so are not publicly available, but may be provided by Natera, Inc. upon reasonable request. Summarized clinical data, sequencing results source data and the original clinical trial protocol are provided as Supplementary materials.

Human research participants

Policy information about studies involving human research participants and Sex and Gender in Research.

Reporting on sex and gender	Patient level information was collected on biological sex and summarized in Table 1. Sex and gender were not considered as covariates for the study design. The study was not designed to capture sex or gender based effects. Sex of participants was determined by self-report.
Population characteristics	Population characteristics including number, sex and age of subjects are summarized in Table 1.
Recruitment	Patients were recruited to and enrolled at a single academic medical center. Trial information was posted to the general public on the institutional webpage as well as governmental website (clinicaltrials.gov). The trial was publicized by presentations at regional and national conferences. The majority of patients recruited were from the surrounding geographic area which may contribute a selection bias.
Ethics oversight	Oversight was provided by the Stanford IRB. All patients provided written, informed consent before enrollment. Participants did not receive compensation.

Note that full information on the approval of the study protocol must also be provided in the manuscript.

Field-specific reporting

Please select the one below that is the best fit for your research. If you are not sure, read the appropriate sections before making your selection.

☒ Life sciences ☐ Behavioural & social sciences ☐ Ecological, evolutionary & environmental sciences

For a reference copy of the document with all sections, see nature.com/documents/nr-reporting-summary-flat.pdf

Life sciences study design

All studies must disclose on these points even when the disclosure is negative.

Sample size	A two stage design was used for enrollment of study participants in Cohort B with a set null hypothesis of 5% objective response rate and alternative response rate of 30% based on standard RECIST v1.1 criteria. Interim analyses were to be performed, separately in each cohort, after accrual of 10 response assessable patients. If at least two out of the 10 patients respond, then 10 additional patients were to be enrolled for a total of 20 patients in each cohort. Based on our statistical constraints, at least 3 patients out of the 20 must respond in each cohort to declare statistical significance at a one sided 5% level with 80% power or better.
Data exclusions	No data were excluded from the study.
Replication	Replication of results was not performed.
Randomization	This was a single arm trial thus randomization was not a component of trial design.
Blinding	Blinding was not relevant to the study design because it was a single arm study.

Reporting for specific materials, systems and methods

We require information from authors about some types of materials, experimental systems and methods used in many studies. Here, indicate whether each material, system or method listed is relevant to your study. If you are not sure if a list item applies to your research, read the appropriate section before selecting a response.

Materials & experimental systems

n/a	Involved in the study	
☒	☐	Antibodies
☒	☐	Eukaryotic cell lines
☒	☐	Palaeontology and archaeology
☒	☐	Animals and other organisms
☐	☒	Clinical data
☒	☐	Dual use research of concern

Methods

n/a	Involved in the study	
☒	☐	ChIP-seq
☒	☐	Flow cytometry
☒	☐	MRI-based neuroimaging

Clinical data

Policy information about clinical studies

All manuscripts should comply with the ICMJE guidelines for publication of clinical research and a completed CONSORT checklist must be included with all submissions.

Clinical trial registration	NCT02401347
Study protocol	The full study protocol is provided with the manuscript submission.
Data collection	Patients were enrolled at Stanford cancer center between August 2015 and December 2019. The final patient discontinued treated in April 2019.
Outcomes	The primary objective was to determine whether single agent talazoparib can result in a 30% or greater rate of objective response. Secondary objectives included determination of the clinical benefit rate (CBR), progression-free survival (PFS) and safety. The primary endpoint was objective response rate based on RECIST 1.1 criteria, which was performed by cross-sectional imaging after every 2 cycles. Tumor measurements were tabulated by the sum of longest diameters of target lesions. The clinical benefit rate is defined as complete response, partial response or stable disease ≥ 24 weeks per RECIST v1.1. Progression free survival was evaluated using the Kaplan Meier method. To assess safety of talazoparib in this study population, adverse events were graded using CTCAE v5 and summarized descriptively.
